# (*E*)-*N*′-(2-Benzyl­oxybenzyl­idene)isonicotinohydrazide methanol solvate monohydrate

**DOI:** 10.1107/S1600536810016958

**Published:** 2010-05-15

**Authors:** H. S. Naveenkumar, Amirin Sadikun, Pazilah Ibrahim, Madhukar Hemamalini, Hoong-Kun Fun

**Affiliations:** a School of Pharmaceutical Sciences, Universiti Sains Malaysia, 11800 USM, Penang, Malaysia; b X-ray Crystallography Unit, School of Physics, Universiti Sains Malaysia, 11800 USM, Penang, Malaysia

## Abstract

The title compound, C_20_H_17_N_3_O_2_·CH_4_O·H_2_O, was synthesized by the condensation reaction of 2-benzyl­oxybenzaldehyde with isoniazid (isonicotinic acid hydrazide). The tricyclic compound displays a *trans* configuration with respect to the C=N double bond. The central benzene ring makes dihedral angles of 8.83 (7) and 70.39 (8)° with the pyridine ring and the terminal benzene ring, respectively. The dihedral angle between the pyridine ring and the terminal benzene ring is 73.11 (8)°. In the crystal structure, mol­ecules are connected by inter­molecular N—H⋯O, O—H⋯O, O—H⋯(N,N) and C—H⋯O hydrogen bonds, forming a two-dimensional network perpendicular to the *a* axis.

## Related literature

For applications of isoniazid derivatives, see: Janin, 2007 ▶; Maccari *et al.* (2005 ▶); Slayden & Barry (2000 ▶). For the biological activity of Schiff bases, see: Kahwa *et al.* (1986 ▶). For related structures, see: Naveenkumar *et al.* (2010*a* ▶, 2010*b* ▶, 2010*c* ▶). For the synthesis of isoniazid derivatives, see: Lourenco *et al.* (2008 ▶). For the stability of the temperature controller used in the data collection, see: Cosier & Glazer (1986 ▶).
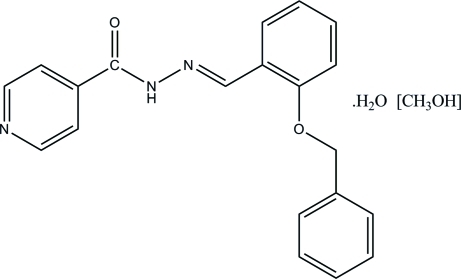


## Experimental

### 

#### Crystal data

C_20_H_17_N_3_O_2_·CH_4_O·H_2_O
*M* *_r_* = 381.42
Monoclinic, 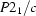

*a* = 17.763 (3) Å
*b* = 12.3888 (18) Å
*c* = 8.7450 (13) Å
β = 98.672 (3)°
*V* = 1902.4 (5) Å^3^
*Z* = 4
Mo *K*α radiation
μ = 0.09 mm^−1^
*T* = 100 K
0.35 × 0.18 × 0.09 mm

#### Data collection

Bruker APEXII DUO CCD area-detector diffractometer
Absorption correction: multi-scan (*SADABS*; Bruker, 2009 ▶) *T*_min_ = 0.968, *T*_max_ = 0.992
24474 measured reflections
6515 independent reflections
4012 reflections with *I* > 2σ(*I*)
*R*_int_ = 0.069

#### Refinement

*R*[*F* ^2^ > 2σ(*F* ^2^)] = 0.059
*wR*(*F* ^2^) = 0.170
*S* = 1.00
6515 reflections
270 parameters
H atoms treated by a mixture of independent and constrained refinement
Δρ_max_ = 0.37 e Å^−3^
Δρ_min_ = −0.35 e Å^−3^

### 

Data collection: *APEX2* (Bruker, 2009 ▶); cell refinement: *SAINT* (Bruker, 2009 ▶); data reduction: *SAINT*; program(s) used to solve structure: *SHELXTL* (Sheldrick, 2008 ▶); program(s) used to refine structure: *SHELXTL*; molecular graphics: *SHELXTL*; software used to prepare material for publication: *SHELXTL* and *PLATON* (Spek, 2009 ▶).

## Supplementary Material

Crystal structure: contains datablocks global, I. DOI: 10.1107/S1600536810016958/sj2796sup1.cif

Structure factors: contains datablocks I. DOI: 10.1107/S1600536810016958/sj2796Isup2.hkl

Additional supplementary materials:  crystallographic information; 3D view; checkCIF report

## Figures and Tables

**Table 1:** Hydrogen-bond geometry (Å, °)

*D*—H⋯*A*	*D*—H	H⋯*A*	*D*⋯*A*	*D*—H⋯*A*
N2—H1*N*2⋯O3	0.91 (2)	2.06 (2)	2.9549 (18)	169.9 (16)
O3—H1*O*3⋯O1*W*	0.87 (3)	1.85 (3)	2.7165 (19)	174 (3)
O1*W*—H1*W*1⋯O1^i^	0.80 (3)	2.11 (3)	2.8713 (18)	160 (2)
O1*W*—H1*W*1⋯N3^i^	0.80 (3)	2.62 (3)	3.2119 (19)	133 (2)
O1*W*—H2*W*1⋯N1^ii^	0.87 (3)	2.05 (3)	2.898 (2)	163 (2)
C1—H1*A*⋯O3	0.93	2.27	3.189 (2)	169
C2—H2*A*⋯O1^iii^	0.93	2.48	3.229 (2)	137

Symmetry codes: (i) 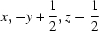; (ii) 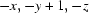; (iii) 
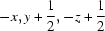
.

**Fig. 1.**
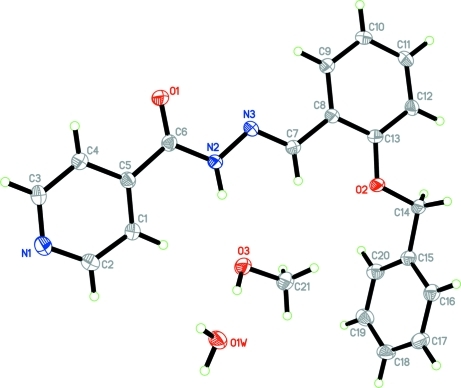
The asymmetric unit of the title compound, showing 50% probability displacement ellipsoids and the atom-numbering scheme.

**Fig. 2.**
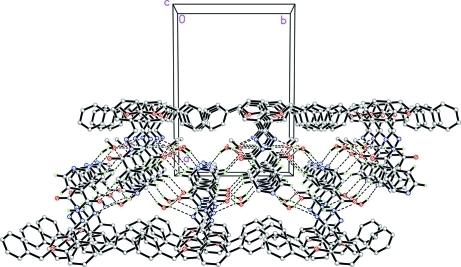
The crystal packing of the title compound, showing the hydrogen-bonding (dashed lines) network.

## supplementary crystallographic information

### Comment

Isoniazid derivatives have been found to possess potential tuberculostatic activity (Janin, 2007; Maccari *et al.*, 2005; Slayden & Barry, 2000). Schiff bases have attracted much attention because of their biological activity (Kahwa *et al.*, 1986).  We have recently reported the crystal structures of (*E*)-*N'*-[(*E*)-3-(4-hydroxy- 3-methoxyphenyl)allylidene]isonicotinohydrazide (Naveenkumar *et al.*, 2010*a*), (*E*)-N'-(2,4,5-trimethoxybenzylidene)isonicotinohydrazide dihydrate (Naveenkumar *et al.*, 2010*b*) and (*E*)-*N'*- (2,4,6-trihydroxybenzylidene)isonicotinohydrazide sesquihydrate (Naveenkumar *et al.*, 2010*c*).  As a part of our current work on the synthesis of (*E*)-*N'*-substituted isonicotinohydrazide derivatives, in this paper we present the crystal structure of the title compound, (I), Fig.1.

In (I), the molecular structure of the compound displays a trans configuration with respect to the C═N double bond.  The central benzene (C8–C13) ring makes dihedral angles of 8.83 (7)° and 70.39 (8)° with the pyridine (N1/C1–C5) ring and the terminal benzene (C15–C20) ring, respectively.  The dihedral angle between the pyridine (N1/C1–C5) ring and the terminal benzene (C15–C20) ring is 73.11 (8)°.

In the crystal packing (Fig. 2), molecules are connected by N2—H1N2···O3, O3—H1O3···O1W, O1W—H1W1···O1, O1W—H1W1···N3, O1W—H2W1···N1, C1—H1A···O3 and C2—H2A···O1 (Table 1) hydrogen bonds.

### Experimental

This isoniazid derivative was prepared by a literature procedure (Lourenco *et al.*, 2008) involving the reaction between the 2-benzyloxybenzaldehyde (1.0 eq) and isoniazid (1.0 eq) in ethanol/water. After stirring for 1-3 hours at room temperature, the resulting mixture was concentrated under reduced pressure. The residue, purified by washing with cold ethanol and diethyl ether, afforded the pure derivative. Colorless single crystals suitable for X-ray analysis were obtained by recrystalization from methanol.

### Refinement

Atoms H1N2, H1O3, H1W1 and H2W1 were located in a difference Fourier map and refined freely. The remaining H atoms were positioned geometrically [C–H = 0.93–0.97 Å] and were refined using a riding model, with *U*_iso_(H) = 1.2 or 1.5*U*_eq_(C).

### Crystal data

**Table:**

C_20_H_17_N_3_O_2_·CH_4_O·H_2_O	*F*(000) = 808
*M**_r_* = 381.42	*D*_x_ = 1.332 Mg m^−^^3^
Monoclinic, *P*2_1_/*c*	Mo *K*α radiation, λ = 0.71073 Å
Hall symbol: -P 2ybc	Cell parameters from 3013 reflections
*a* = 17.763 (3) Å	θ = 2.3–30.0°
*b* = 12.3888 (18) Å	µ = 0.09 mm^−^^1^
*c* = 8.7450 (13) Å	*T* = 100 K
β = 98.672 (3)°	Plate, colourless
*V* = 1902.4 (5) Å^3^	0.35 × 0.18 × 0.09 mm
*Z* = 4	

### Data collection

**Table:**

Bruker APEXII DUO CCD area-detector diffractometer	6515 independent reflections
Radiation source: fine-focus sealed tube	4012 reflections with *I* > 2σ(*I*)
graphite	*R*_int_ = 0.069
φ and ω scans	θ_max_ = 32.0°, θ_min_ = 2.0°
Absorption correction: multi-scan (*SADABS*; Bruker, 2009)	*h* = −26→26
*T*_min_ = 0.968, *T*_max_ = 0.992	*k* = −17→18
24474 measured reflections	*l* = −13→12

### Refinement

**Table:**

Refinement on *F*^2^	Primary atom site location: structure-invariant direct methods
Least-squares matrix: full	Secondary atom site location: difference Fourier map
*R*[*F*^2^ > 2σ(*F*^2^)] = 0.059	Hydrogen site location: inferred from neighbouring sites
*wR*(*F*^2^) = 0.170	H atoms treated by a mixture of independent and constrained refinement
*S* = 1.00	*w* = 1/[σ^2^(*F*_o_^2^) + (0.0887*P*)^2^] where *P* = (*F*_o_^2^ + 2*F*_c_^2^)/3
6515 reflections	(Δ/σ)_max_ < 0.001
270 parameters	Δρ_max_ = 0.37 e Å^−^^3^
0 restraints	Δρ_min_ = −0.35 e Å^−^^3^

### Special details

**Table:**

Experimental. The crystal was placed in the cold stream of an Oxford Cryosystems Cobra open-flow nitrogen cryostat (Cosier & Glazer, 1986) operating at 100.0 (1) K.
Geometry. All s.u.'s (except the s.u. in the dihedral angle between two l.s. planes) are estimated using the full covariance matrix. The cell s.u.'s are taken into account individually in the estimation of s.u.'s in distances, angles and torsion angles; correlations between s.u.'s in cell parameters are only used when they are defined by crystal symmetry. An approximate (isotropic) treatment of cell s.u.'s is used for estimating s.u.'s involving l.s. planes.
Refinement. Refinement of F^2^ against ALL reflections. The weighted R-factor wR and goodness of fit S are based on F^2^, conventional R-factors R are based on F, with F set to zero for negative F^2^. The threshold expression of F^2^ > 2σ(F^2^) is used only for calculating R-factors(gt) etc. and is not relevant to the choice of reflections for refinement. R-factors based on F^2^ are statistically about twice as large as those based on F, and R- factors based on ALL data will be even larger.

### Fractional atomic coordinates and isotropic or equivalent isotropic displacement parameters (Å^2^)

**Table:**

	*x*	*y*	*z*	*U*_iso_*/*U*_eq_	
O1	0.12066 (6)	0.05407 (9)	0.36337 (14)	0.0250 (3)	
O2	0.36364 (6)	0.37324 (8)	0.80611 (13)	0.0211 (2)	
N1	−0.05513 (8)	0.28776 (12)	0.00600 (16)	0.0258 (3)	
N2	0.17041 (7)	0.21885 (11)	0.43066 (15)	0.0194 (3)	
N3	0.22141 (7)	0.17181 (10)	0.54646 (15)	0.0196 (3)	
C1	0.04882 (9)	0.31412 (13)	0.2126 (2)	0.0241 (3)	
H1A	0.0791	0.3622	0.2765	0.029*	
C2	−0.00980 (9)	0.35168 (14)	0.1031 (2)	0.0270 (4)	
H2A	−0.0181	0.4258	0.0966	0.032*	
C3	−0.04163 (10)	0.18181 (14)	0.0186 (2)	0.0284 (4)	
H3A	−0.0721	0.1356	−0.0480	0.034*	
C4	0.01531 (9)	0.13683 (13)	0.1257 (2)	0.0256 (3)	
H4A	0.0223	0.0624	0.1304	0.031*	
C5	0.06174 (8)	0.20416 (12)	0.22571 (17)	0.0191 (3)	
C6	0.12010 (8)	0.15226 (12)	0.34544 (18)	0.0194 (3)	
C7	0.26917 (8)	0.23667 (12)	0.62477 (18)	0.0192 (3)	
H7A	0.2676	0.3101	0.6022	0.023*	
C8	0.32580 (8)	0.19465 (12)	0.74905 (17)	0.0176 (3)	
C9	0.33274 (8)	0.08431 (12)	0.78078 (18)	0.0200 (3)	
H9A	0.3004	0.0360	0.7218	0.024*	
C10	0.38701 (9)	0.04540 (12)	0.89869 (19)	0.0216 (3)	
H10A	0.3906	−0.0282	0.9193	0.026*	
C11	0.43586 (8)	0.11734 (13)	0.98564 (19)	0.0216 (3)	
H11A	0.4724	0.0914	1.0644	0.026*	
C12	0.43089 (8)	0.22761 (12)	0.95664 (18)	0.0198 (3)	
H12A	0.4644	0.2751	1.0145	0.024*	
C13	0.37506 (8)	0.26656 (11)	0.83943 (17)	0.0179 (3)	
C14	0.40996 (9)	0.45051 (12)	0.90037 (19)	0.0212 (3)	
H14A	0.4624	0.4457	0.8825	0.025*	
H14B	0.4084	0.4369	1.0091	0.025*	
C15	0.37824 (9)	0.55991 (12)	0.85576 (17)	0.0201 (3)	
C16	0.41662 (9)	0.63173 (13)	0.77156 (19)	0.0240 (3)	
H16A	0.4638	0.6129	0.7455	0.029*	
C17	0.38469 (10)	0.73098 (13)	0.7266 (2)	0.0291 (4)	
H17A	0.4104	0.7784	0.6702	0.035*	
C18	0.31456 (10)	0.75978 (13)	0.7654 (2)	0.0287 (4)	
H18A	0.2932	0.8263	0.7349	0.034*	
C19	0.27639 (10)	0.68912 (14)	0.8500 (2)	0.0278 (4)	
H19A	0.2295	0.7086	0.8768	0.033*	
C20	0.30781 (9)	0.58952 (13)	0.89466 (19)	0.0245 (3)	
H20A	0.2818	0.5423	0.9508	0.029*	
O3	0.17234 (7)	0.45730 (9)	0.42445 (15)	0.0268 (3)	
C21	0.21264 (10)	0.52275 (14)	0.5437 (2)	0.0269 (3)	
H21A	0.2657	0.5039	0.5583	0.040*	
H21B	0.1926	0.5110	0.6383	0.040*	
H21C	0.2068	0.5974	0.5149	0.040*	
O1W	0.16524 (7)	0.56017 (11)	0.14832 (15)	0.0261 (3)	
H1N2	0.1707 (11)	0.2913 (16)	0.416 (2)	0.027 (5)*	
H1O3	0.1722 (13)	0.4936 (19)	0.339 (3)	0.051 (7)*	
H1W1	0.1606 (14)	0.518 (2)	0.079 (3)	0.052 (7)*	
H2W1	0.1375 (15)	0.616 (2)	0.115 (3)	0.056 (7)*	

### Atomic displacement parameters (Å^2^)

**Table:**

	*U*^11^	*U*^22^	*U*^33^	*U*^12^	*U*^13^	*U*^23^
O1	0.0287 (6)	0.0175 (5)	0.0262 (6)	−0.0017 (4)	−0.0046 (5)	0.0026 (5)
O2	0.0244 (5)	0.0139 (5)	0.0223 (5)	−0.0010 (4)	−0.0054 (4)	−0.0011 (4)
N1	0.0228 (6)	0.0274 (7)	0.0257 (7)	0.0022 (5)	−0.0009 (6)	0.0010 (6)
N2	0.0213 (6)	0.0161 (6)	0.0187 (6)	−0.0002 (5)	−0.0032 (5)	0.0025 (5)
N3	0.0188 (6)	0.0188 (6)	0.0199 (6)	0.0012 (4)	−0.0016 (5)	0.0024 (5)
C1	0.0232 (7)	0.0209 (7)	0.0259 (8)	−0.0017 (6)	−0.0038 (6)	−0.0008 (7)
C2	0.0259 (8)	0.0224 (8)	0.0309 (9)	0.0036 (6)	−0.0012 (7)	0.0024 (7)
C3	0.0274 (8)	0.0283 (9)	0.0259 (8)	−0.0019 (6)	−0.0078 (7)	−0.0013 (7)
C4	0.0275 (8)	0.0196 (8)	0.0269 (8)	−0.0007 (6)	−0.0045 (7)	−0.0009 (7)
C5	0.0181 (6)	0.0209 (7)	0.0179 (7)	−0.0005 (5)	0.0012 (6)	0.0020 (6)
C6	0.0203 (7)	0.0200 (7)	0.0173 (7)	−0.0019 (5)	0.0011 (6)	0.0000 (6)
C7	0.0207 (7)	0.0156 (7)	0.0202 (7)	−0.0008 (5)	−0.0008 (6)	0.0008 (6)
C8	0.0181 (6)	0.0165 (7)	0.0177 (7)	0.0006 (5)	0.0005 (5)	0.0012 (6)
C9	0.0205 (7)	0.0174 (7)	0.0213 (7)	−0.0007 (5)	0.0006 (6)	−0.0015 (6)
C10	0.0231 (7)	0.0163 (7)	0.0249 (8)	0.0022 (5)	0.0020 (6)	0.0035 (6)
C11	0.0195 (7)	0.0233 (8)	0.0208 (7)	0.0039 (5)	−0.0006 (6)	0.0025 (6)
C12	0.0178 (6)	0.0209 (7)	0.0195 (7)	0.0003 (5)	−0.0010 (6)	−0.0012 (6)
C13	0.0197 (6)	0.0152 (7)	0.0190 (7)	0.0011 (5)	0.0032 (6)	−0.0001 (6)
C14	0.0225 (7)	0.0170 (7)	0.0222 (7)	−0.0024 (5)	−0.0032 (6)	−0.0014 (6)
C15	0.0252 (7)	0.0170 (7)	0.0165 (7)	−0.0015 (5)	−0.0022 (6)	−0.0019 (6)
C16	0.0245 (7)	0.0228 (8)	0.0234 (8)	−0.0026 (6)	−0.0003 (6)	−0.0005 (7)
C17	0.0339 (9)	0.0220 (8)	0.0294 (9)	−0.0054 (6)	−0.0013 (7)	0.0039 (7)
C18	0.0361 (9)	0.0174 (8)	0.0294 (9)	0.0018 (6)	−0.0053 (8)	−0.0013 (7)
C19	0.0300 (8)	0.0251 (8)	0.0270 (8)	0.0051 (6)	0.0003 (7)	−0.0035 (7)
C20	0.0291 (8)	0.0222 (8)	0.0220 (8)	−0.0002 (6)	0.0032 (7)	−0.0003 (7)
O3	0.0307 (6)	0.0224 (6)	0.0244 (6)	−0.0056 (4)	−0.0052 (5)	0.0018 (5)
C21	0.0288 (8)	0.0243 (8)	0.0262 (8)	−0.0023 (6)	0.0000 (7)	−0.0033 (7)
O1W	0.0298 (6)	0.0224 (6)	0.0231 (6)	0.0033 (5)	−0.0058 (5)	−0.0012 (5)

### Geometric parameters (Å, °)

**Table:**

O1—C6	1.2263 (18)	C11—C12	1.390 (2)
O2—C13	1.3619 (17)	C11—H11A	0.9300
O2—C14	1.4382 (18)	C12—C13	1.400 (2)
N1—C3	1.336 (2)	C12—H12A	0.9300
N1—C2	1.338 (2)	C14—C15	1.497 (2)
N2—C6	1.3542 (19)	C14—H14A	0.9700
N2—N3	1.3817 (18)	C14—H14B	0.9700
N2—H1N2	0.91 (2)	C15—C20	1.394 (2)
N3—C7	1.2881 (19)	C15—C16	1.397 (2)
C1—C5	1.383 (2)	C16—C17	1.386 (2)
C1—C2	1.385 (2)	C16—H16A	0.9300
C1—H1A	0.9300	C17—C18	1.386 (3)
C2—H2A	0.9300	C17—H17A	0.9300
C3—C4	1.387 (2)	C18—C19	1.387 (2)
C3—H3A	0.9300	C18—H18A	0.9300
C4—C5	1.387 (2)	C19—C20	1.386 (2)
C4—H4A	0.9300	C19—H19A	0.9300
C5—C6	1.502 (2)	C20—H20A	0.9300
C7—C8	1.461 (2)	O3—C21	1.426 (2)
C7—H7A	0.9300	O3—H1O3	0.88 (2)
C8—C9	1.397 (2)	C21—H21A	0.9600
C8—C13	1.406 (2)	C21—H21B	0.9600
C9—C10	1.388 (2)	C21—H21C	0.9600
C9—H9A	0.9300	O1W—H1W1	0.80 (3)
C10—C11	1.388 (2)	O1W—H2W1	0.88 (3)
C10—H10A	0.9300		
			
C13—O2—C14	117.99 (12)	C11—C12—C13	119.40 (14)
C3—N1—C2	116.47 (15)	C11—C12—H12A	120.3
C6—N2—N3	116.85 (13)	C13—C12—H12A	120.3
C6—N2—H1N2	123.0 (12)	O2—C13—C12	123.87 (14)
N3—N2—H1N2	120.1 (12)	O2—C13—C8	115.77 (13)
C7—N3—N2	115.66 (13)	C12—C13—C8	120.35 (13)
C5—C1—C2	119.11 (15)	O2—C14—C15	107.03 (12)
C5—C1—H1A	120.4	O2—C14—H14A	110.3
C2—C1—H1A	120.4	C15—C14—H14A	110.3
N1—C2—C1	123.90 (15)	O2—C14—H14B	110.3
N1—C2—H2A	118.0	C15—C14—H14B	110.3
C1—C2—H2A	118.0	H14A—C14—H14B	108.6
N1—C3—C4	123.64 (16)	C20—C15—C16	119.24 (14)
N1—C3—H3A	118.2	C20—C15—C14	119.41 (14)
C4—C3—H3A	118.2	C16—C15—C14	121.32 (14)
C5—C4—C3	119.19 (15)	C17—C16—C15	120.22 (15)
C5—C4—H4A	120.4	C17—C16—H16A	119.9
C3—C4—H4A	120.4	C15—C16—H16A	119.9
C1—C5—C4	117.68 (14)	C16—C17—C18	120.22 (16)
C1—C5—C6	124.60 (14)	C16—C17—H17A	119.9
C4—C5—C6	117.66 (14)	C18—C17—H17A	119.9
O1—C6—N2	122.82 (14)	C17—C18—C19	119.84 (15)
O1—C6—C5	120.34 (14)	C17—C18—H18A	120.1
N2—C6—C5	116.84 (13)	C19—C18—H18A	120.1
N3—C7—C8	119.84 (13)	C20—C19—C18	120.24 (16)
N3—C7—H7A	120.1	C20—C19—H19A	119.9
C8—C7—H7A	120.1	C18—C19—H19A	119.9
C9—C8—C13	118.68 (13)	C19—C20—C15	120.24 (15)
C9—C8—C7	121.79 (13)	C19—C20—H20A	119.9
C13—C8—C7	119.53 (13)	C15—C20—H20A	119.9
C10—C9—C8	121.22 (14)	C21—O3—H1O3	105.8 (16)
C10—C9—H9A	119.4	O3—C21—H21A	109.5
C8—C9—H9A	119.4	O3—C21—H21B	109.5
C9—C10—C11	119.40 (14)	H21A—C21—H21B	109.5
C9—C10—H10A	120.3	O3—C21—H21C	109.5
C11—C10—H10A	120.3	H21A—C21—H21C	109.5
C10—C11—C12	120.93 (14)	H21B—C21—H21C	109.5
C10—C11—H11A	119.5	H1W1—O1W—H2W1	107 (2)
C12—C11—H11A	119.5		
			
C6—N2—N3—C7	179.30 (13)	C9—C10—C11—C12	0.3 (2)
C3—N1—C2—C1	0.1 (2)	C10—C11—C12—C13	0.9 (2)
C5—C1—C2—N1	−0.8 (3)	C14—O2—C13—C12	−2.2 (2)
C2—N1—C3—C4	0.5 (2)	C14—O2—C13—C8	176.90 (12)
N1—C3—C4—C5	−0.4 (3)	C11—C12—C13—O2	177.34 (13)
C2—C1—C5—C4	0.9 (2)	C11—C12—C13—C8	−1.7 (2)
C2—C1—C5—C6	−176.15 (14)	C9—C8—C13—O2	−177.83 (12)
C3—C4—C5—C1	−0.3 (2)	C7—C8—C13—O2	2.38 (19)
C3—C4—C5—C6	176.90 (14)	C9—C8—C13—C12	1.3 (2)
N3—N2—C6—O1	−3.1 (2)	C7—C8—C13—C12	−178.47 (13)
N3—N2—C6—C5	176.55 (12)	C13—O2—C14—C15	−171.52 (12)
C1—C5—C6—O1	169.54 (15)	O2—C14—C15—C20	69.93 (18)
C4—C5—C6—O1	−7.5 (2)	O2—C14—C15—C16	−107.99 (16)
C1—C5—C6—N2	−10.1 (2)	C20—C15—C16—C17	−0.4 (2)
C4—C5—C6—N2	172.90 (14)	C14—C15—C16—C17	177.56 (15)
N2—N3—C7—C8	−179.56 (12)	C15—C16—C17—C18	0.2 (3)
N3—C7—C8—C9	3.6 (2)	C16—C17—C18—C19	0.2 (3)
N3—C7—C8—C13	−176.60 (13)	C17—C18—C19—C20	−0.5 (3)
C13—C8—C9—C10	−0.1 (2)	C18—C19—C20—C15	0.4 (3)
C7—C8—C9—C10	179.69 (14)	C16—C15—C20—C19	0.1 (2)
C8—C9—C10—C11	−0.7 (2)	C14—C15—C20—C19	−177.91 (15)

### Hydrogen-bond geometry (Å, °)

**Table:**

*D*—H···*A*	*D*—H	H···*A*	*D*···*A*	*D*—H···*A*
N2—H1N2···O3	0.91 (2)	2.06 (2)	2.9549 (18)	169.9 (16)
O3—H1O3···O1W	0.87 (3)	1.85 (3)	2.7165 (19)	174 (3)
O1W—H1W1···O1^i^	0.80 (3)	2.11 (3)	2.8713 (18)	160 (2)
O1W—H1W1···N3^i^	0.80 (3)	2.62 (3)	3.2119 (19)	133 (2)
O1W—H2W1···N1^ii^	0.87 (3)	2.05 (3)	2.898 (2)	163 (2)
C1—H1A···O3	0.93	2.27	3.189 (2)	169
C2—H2A···O1^iii^	0.93	2.48	3.229 (2)	137

Symmetry codes: (i) *x*, −*y*+1/2, *z*−1/2; (ii) −*x*, −*y*+1, −*z*; (iii) −*x*, *y*+1/2, −*z*+1/2.
